# Integrated Intelligent Method Based on Fuzzy Logic for Optimizing Laser Microfabrication Processing of GnPs-Improved Alumina Nanocomposites

**DOI:** 10.3390/mi14040750

**Published:** 2023-03-29

**Authors:** Khaled N. Alqahtani, Mustafa M. Nasr, Saqib Anwar, Ali M. Al-Samhan, Mohammed H. Alhaag, Husam Kaid

**Affiliations:** 1 Industrial Engineering Department, College of Engineering, Taibah University, Medina 41411, Saudi Arabia; 2 Industrial Engineering Department, College of Engineering, King Saud University, Riyadh 11421, Saudi Arabia

**Keywords:** alumina bioceramic nanocomposites, graphene nanoplatelets, laser micromachining performance, artificial intelligence, ANFIS technique, MOPSO approach, material removal rate

## Abstract

Studies on using multifunctional graphene nanostructures to enhance the microfabrication processing of monolithic alumina are still rare and too limited to meet the requirements of green manufacturing criteria. Therefore, this study aims to increase the ablation depth and material removal rate and minimize the roughness of the fabricated microchannel of alumina-based nanocomposites. To achieve this, high-density alumina nanocomposites with different graphene nanoplatelet (GnP) contents (0.5 wt.%, 1 wt.%, 1.5 wt.%, and 2.5 wt.%) were fabricated. Afterward, statistical analysis based on the full factorial design was performed to study the influence of the graphene reinforcement ratio, scanning speed, and frequency on material removal rate (MRR), surface roughness, and ablation depth during low-power laser micromachining. After that, an integrated intelligent multi-objective optimization approach based on the adaptive neuro-fuzzy inference system (ANIFS) and multi-objective particle swarm optimization approach was developed to monitor and find the optimal GnP ratio and microlaser parameters. The results reveal that the GnP reinforcement ratio significantly affects the laser micromachining performance of Al_2_O_3_ nanocomposites. This study also revealed that the developed ANFIS models could obtain an accurate estimation model for monitoring the surface roughness, MRR, and ablation depth with fewer errors than 52.07%, 100.15%, and 76% for surface roughness, MRR, and ablation depth, respectively, in comparison with the mathematical models. The integrated intelligent optimization approach indicated that a GnP reinforcement ratio of 2.16, scanning speed of 342 mm/s, and frequency of 20 kHz led to the fabrication of microchannels with high quality and accuracy of Al_2_O_3_ nanocomposites. In contrast, the unreinforced alumina could not be machined using the same optimized parameters with low-power laser technology. Henceforth, an integrated intelligence method is a powerful tool for monitoring and optimizing the micromachining processes of ceramic nanocomposites, as demonstrated by the obtained results.

## 1. Introduction

In the recently published literature, ceramic matrix nanocomposites (CMCs) are particularly interesting since they exhibit enhancements in mechanical properties and thermal and electrical conductivity compared to monolith ceramics [1,2,3,4,5,6]. Many engineering components are fabricated from ceramic and ceramic matrix composites using different near-net shape manufacturing technologies. Therefore, the machining of these materials is fundamentally required to achieve the desired surface finish, dimensional accuracy, and form accuracy to satisfy functional requirements [7]. However, machining ceramic matrix nanocomposites reinforced with fibers is very difficult compared to non-reinforced materials. This is due to hard phases in composites and the weak interface, which causes poor surface morphology and rapid tool wear during machining [6,8]. Therefore, poor machinability and high machining costs limit their use in the industry. Hence, it has become essential to address certain issues to enhance the machining properties of ceramic matrix nanocomposites to increase their use in different engineering applications. It is expected that these may be overcome by adding graphene reinforcement to the ceramic matrix due to the excellent properties of graphene, such as mechanical, thermal, and electrical properties [9,10]. In addition, it has advantages such as less tendency to tangle and a higher specific surface area, making it easier to disperse into nanomaterials than other reinforcement materials [10].

Recently, graphene is becoming an increasingly attractive nanofiller material for enhancing ceramic nanocomposite performance. Graphene/alumina matrix nanocomposites are examples of ceramic matrix nanocomposites that exhibit high biocompatibility, strength, elastic stiffness, and stability compared with monolith alumina. These properties make them a good choice for automotive, aerospace, and biomedical applications [11]. Alumina/graphene nanocomposites with varying graphene contents have been successfully produced through powder metallurgy technologies such as high-frequency induction heating (HFIHs), hot pressing (HP), spark plasma sintering (SPS), and hot isostatic pressing (HIP). Many studies have focused on enhancements in the properties of the Al_2_O_3-_based nanocomposites after adding the graphene reinforcement material [12,13,14,15,16,17,18,19,20,21,22]. Thereafter, micromachining becomes essential after their fabrication to meet the requirements of the desired application, either as micro components or products. However, pure alumina materials are very difficult to cut, i.e., the micromachining of these materials is also very challenging.

Improving the micromachining of the materials and their composites can be divided into developing a hybrid machining process, designing a new tool, optimizing machining parameters, and nano-reinforcement materials and their ratio. Research on machining ceramic and ceramic matrix composites has mainly focused on optimizing machining parameters and designing new tools. For instance, Bertsche et al. [23] studied the effect of diamond tool characteristics on the cutting forces, surface roughness, and tool wear during rotary ultrasonic machining of silicon carbide matrix composite. They found that hard diamond grains, grain size, and diamond concentration significantly affect the surface quality, cutting force, and tool wear. Wang et al. [24] developed a novel step-taper diamond core drill for rotary ultrasonic machining of silicon carbide matrix composite to improve the hole exit quality. Liu et al. [25] investigated the influence of energy density and feeding speed on the quality of SiC/SiC composite micro-holes using a picosecond laser. The results show that feeding speed and energy density affected the micro-hole quality. Zhai et al. [26] used a high-repetition frequency femtosecond laser to machine SiC/SiC composites. They discussed the influence of the pulsed laser on the surface microstructure and formation conditions. They successfully controlled the surface oxidation of the SiC/SiC and achieved good morphology by optimizing laser parameters.

Ultrafast laser is becoming an increasingly common technology in the microfabrication processing of alumina ceramics due to its high-precision accuracy. For instance, Mohammed et al. [27] investigated the influence of pulse overlap and laser fluence on microchannels of alumina ceramic using an Nd: YAG laser. They found that the fabricated microchannels with moderate pulse overlap exhibited good quality compared to low pulse overlaps. Zhang et al. [28] used picosecond laser technology to achieve high-precision surface polishing of Al_2_O_3_ ceramics. Their study showed that surface roughness after polishing was 82% lower than that of unpolished samples. Esmail et al. [29] used a picosecond laser to fabricate cavities on alumina ceramics. They studied the effect of wobble frequency, wobble amplitude, wobble pitch, and linear speed on the ablation depth, surface roughness, and defect-free cuts of desired geometries with high precision. The results show that deeper cuts and smaller kerf tapers are produced by smaller wobble amplitudes and lower frequencies. In addition, surface roughness increased significantly for wobble pitches above 30 µm. Preusch et al. [30] used a high-precision fiber laser to fabricate microchannels on alumina ceramics to investigate the effect of pulse overlap and laser repetition rate on the surface roughness, material removal rate, and dimensional accuracy. They found that a minimum roughness of 1.5 μm for alumina ceramics could be obtained when the pulse overlap was 42%. Jia et al. [31] developed a numerical model of a combined pulse laser to improve the drilling efficiency of alumina ceramics. However, these ultrafast laser techniques still have several drawbacks, such as high cost, low efficiency, and large damage to the substrate [32].

It has been reported that very limited work has been carried out on improving the micromachining of alumina ceramic using nanofillers such as graphene. For instance, Sung et al. [33] reported that graphene-reinforced aluminum oxide increased the electrical conductivity of the fabricated composites. Additionally, they used electrical discharge machining (EDM) to evaluate the effect of adding graphene on the machined surface of the developed materials. Kim et al. [34] used the femtosecond laser technique to micromachine CNT/alumina nanocomposites. It was found that adding the CNT to Al_2_O_3_ enhanced the machining of these new materials due to their excellent properties, which led to higher thermal conductivity, lower light transmittance, and suppressed grain growth. Le et al. [35] investigated the influence of graphene nanostructure and carbon nanotubes on thermal conductivity and optical absorbance. It was found that higher optical absorbance and thermal conductivity of CNT/Al_2_O_3_ composite and GnPs/Al_2_O_3_ composite resulted in a lower ablation threshold, leading to an increase in the ablation depth. However, no information about the optimal reinforcement ratio is available, which enhances the ablation depth and quality. Therefore, there is a need to optimize the reinforcement ratio, which improves the quality of micromachining and MRR and reduces power consumption.

It is important to emphasize that choosing optimal microfabrication and machining conditions of a graphene-based ceramic matrix composite plays a crucial role in environmentally friendly and energy-efficient manufacturing. In addition, it ensures the quality of microfabrication components, lowering manufacturing costs and enhancing productivity. Therefore, several traditional techniques have been proposed for optimizing the machining conditions, such as the response surface method (RSM) [36,37] and Taguchi method [38]. However, these techniques depend on the randomly chosen initial solutions, and the optimal solutions fall into the local solution [39,40]. Several metaheuristic algorithms have been developed to guarantee optimal global solutions for micro/macro machining properties (such as genetic algorithms and multi-objective particle swarm optimizations). For instance, Jiang et al. [41] presented a GA for optimizing the machining conditions during turning TiB_2_-based aluminum to minimize surface finish and maximize production time. Cupta [42] used PSO and RSM to optimize the turning parameters for reducing the roughness of the machined surface, tool wear, and cutting forces, using the PSO method response surface method. Hybrid metaheuristic algorithms such as PSO and GA were proposed by the authors of [43] for optimizing arc welding process parameters. In addition, some researchers adapted the MOPSO method to overcome the limitations of the GA method, such as the computation time being longer, too many control parameters, and the convergence being deliberate [39]. MOPSO is faster than GA and can perform global and local searches simultaneously, whereas GA is primarily effective for global search, as reported by the authors of [39,42,44,45]. The prediction models determine the effectiveness of optimization methods as fitness functions. These models were developed using regression analysis, RSM, and factorial design, which cannot guarantee reliable results of the macro/micromachining processes because these processes are very complex and exhibit nonlinear behaviors. As a result, there is growing interest in developing models for macro/micromachining to ensure reliable results. Artificial intelligence approaches are powerful tools for modeling complex nonlinear systems [46,47].

It has been shown that adaptive neuro-fuzzy inference systems (ANFIS) provide more realistic results than artificial neural networks (ANN) and mathematical models [48,49,50,51]. In addition, integrating the ANFIS approach with optimization methods as a fitness function provided accurate results compared to the Taguchi and RSM models. Conde et al. [47] combined artificial neural networks and simulated annealing to optimize the EDM. Gopan et al. [39] optimized the grinding conditions using ANN and PSO methods for reducing the cutting forces and surface roughness, and obtained accurate results during validation. Abbas et al. [52] combined ANN with the Edgeworth–Pareto method to optimize face milling parameters to minimize machining time and surface roughness. Nasr et al. [53] developed a new integrated approach based on ANFIS and MOPSO to optimize fabrication parameters. Comparing the results of this integrated approach with those of the traditional (desirability method), they showed it to be more accurate. Moreover, this integrated approach needs more investigation in optimizing machining parameters.

According to the reviewed literature, it is evident that graphene nano-reinforcement materials enhance ceramic nanocomposites’ machinability and functionality. It was also found that studies on the influence of the macro/micromachining parameters and graphene reinforcement ratio on enhancing the microfabrication performance of Al_2_O_3_ ceramics are still too limited and incomplete to meet the requirements of green manufacturing [54,55]. There has been no previous work reported in the literature on optimizing microfabrication processing for GnP-improved alumina ceramic nanocomposites. Only one study [35] reported on the effect of graphene on optical absorbance and thermal conductivity with ablation characteristics. The authors found that despite improved optical absorbance, graphene-reinforced Al_2_O_3_ matrix nanocomposites exhibited improvement in micromachining depth. Despite this, their work did not consider GnP reinforcement ratios and microlaser parameters for improving micromachining performance, such as MRR, surface roughness, and accuracy. Additionally, they used highly expensive laser technology for testing the machinability of graphene-based nanocomposites.

This work aims to increase the ablation depth and material removal rate and minimize the roughness of the fabricated microchannel of alumina-based nanocomposites to enhance their machinability. To achieve this objective, firstly, high-density GnP-reinforced Al_2_O_3_-based nanocomposites with different GnP contents were produced by using the ball mill and HFIHs processes. Secondly, laser microchannel experiments were conducted using a full factorial design to explore the influence of scanning speed, frequency, and GnP ratio on laser microchannels on MRR, surface roughness, and microchannel accuracy. Thirdly, artificial intelligence models based on the ANFIS technique were developed for monitoring the micromachining performance. Finally, integrated intelligent ANFIS models with the MOPSO approach were developed to obtain the optimal GnP reinforcement ratio and microlaser parameters that enhance the micromachining quality, production time, and accuracy of the fabricated microchannels.

## 2. Experimental Methods

### 2.1. Fabrication of the Nanocomposites

The chemical composition of the purchased Al_2_O_3_ (US Research Nanomaterials, Inc., Houston, TX, USA) is presented in Table 1. Al_2_O_3_ matrix nanocomposites reinforced with different weight percentages of GnPs (0.5 wt.%, 1 wt.%, 1.5 wt.%, and 2.5 wt.%) were produced. The graphene used in this study was GnPs-C-750 from XG Sciences, Inc. East Lansing, MI, USA. Their average surface area of 750 m^2^/g and nominal diameter of less than 2 µm characterize the GnPs, which are a few nanometers thick (5–8 nm).

**Figure 1:**
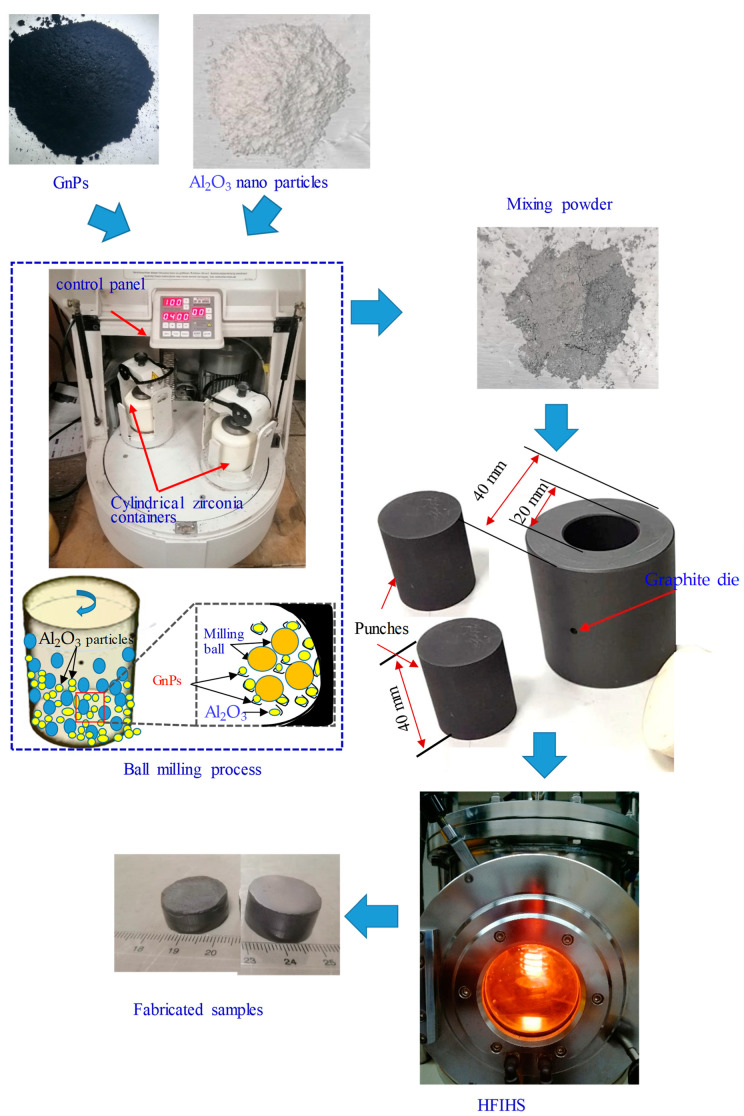
Schematic illustration of producing specimens and fabrication setup.

A Pulverisette ball mill machine blended all GnPs into Al_2_O_3_ particles. The milling process was carried out for 4 h at 350 rpm with a ball-to-powder weight of 20:1 [56]. As a result of the ball-milling process, the powder was placed into a graphite die with an outer diameter of 40 mm and an interior diameter of 20 mm, which was then sintered using HFIHs technology. The sintering was performed at a heating rate of 200 °C/min, a temperature of 1350 °C, and a uniaxial pressure of 60 MPa [57]. The flow diagram of the production procedure of the nanocomposite specimens is shown in Figure 1. The dimensions of the fabricated samples were a diameter of 20 mm and a height of 15 mm. After fabrication, all specimens were ground using silicon carbide papers ranging between P800 and P2500 grit sizes. Then, the actual density of the produced nanocomposite samples was measured by employing the Archimedes method to evaluate the manufactured specimens. The theoretical density of the nanocomposites was calculated. Later, the relative density was computed from the ratio of the actual measured density to the calculated theoretical density. The fabricated specimens presented high relative densities ranging from 0.995 to 0.972, which indicates good bonding between the GnPs and alumina ceramic particles. A Vickers hardness tester ZHV30 was used to measure the hardness of the produced specimens by using a load of 30 Kg for a dwell time of 12 s. A seven-time hardness measurement was conducted for each specimen at different locations on the polished surface, and then the average value was calculated, as shown in Table 2. It can be concluded that the sample with low GnP content exhibited the highest hardness compared with other GnP specimens. This is in agreement with previous studies [18,20].

### 2.2. Micromachining Setup and Measurements

**Figure 2:**
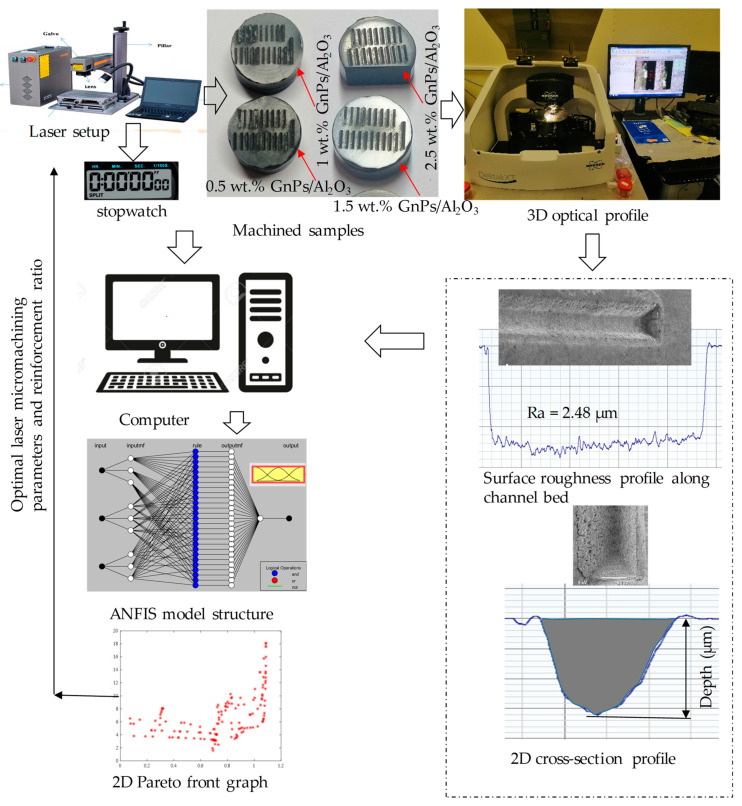
Schematic illustration of integrated intelligent system with experimental setup and measuring devices.

The laser micro-machining process was carried out using a fiber laser (XTL-FP 20, XT laser, Shandong, China). The laser beam is focused using a flat-field lens, moved through a galvanometric mirror system, and irradiated on the top surface of all fabricated samples. Microchannels 250 µm (micron) in width and 5 mm in length were fabricated on all produced GnPs/Al_2_O_3_ nanocomposites with various graphene contents. After repeating each experiment twice, average measurements were calculated. A 3D optical profilometer (DektakXT Stylus Profiler) from Bruker (Billerica, MA, USA) was used to measure dimensional accuracy (D) and surface roughness (SR). Measurements of the ablated depth were made by capturing four random 2D profiles across the channel width. The average of the measured 2D profiles was used later to measure the dimensional accuracy, as shown in Figure 2. Roughness was measured as arithmetic mean surface roughness (Ra) according to the ISO 4287 standard. The SR was measured by scanning three random regions along the channel’s bottom length and averaging them for further analysis. To evaluate how the graphene content affects the laser microfabrication processing of GnPs/Al_2_O_3_ nanocomposites, the MRR was computed using Equation (1).

(1) $$MRR=\frac{Cross-sectional\;area\times channel\;length}{micromachining\;time}({mm}^{3}/min)$$

The micromachined area along the cross-section was calculated from the fitted 2D profile, as shown in Figure 2.

### 2.3. Experimental Design

**Figure 3:**
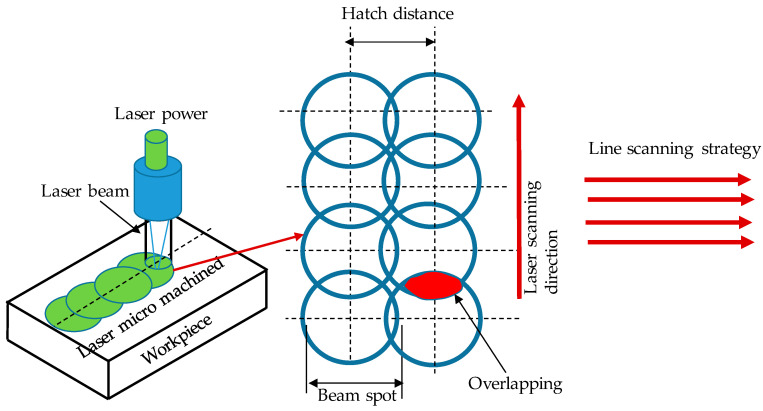
Graphical representation of the laser ablation track and line scanning strategy.

The experiments were conducted based on full factorial design conditions, and the effect of the GnP reinforcement ratio and laser parameters on the output responses was evaluated. The microchannels were machined on all fabricated GnPs/Al_2_O_3_ nanocomposites with varying graphene contents. Two laser parameters, scanning speed and pulse frequency, were selected and changed during the micromachining. No information is available in the literature regarding the laser micromachining of GnP/Al_2_O_3_-based matrix nanocomposites. Therefore, preliminary tests were initially performed to find the suitable range of influential factors. The selected micromachining parameters, GnP reinforcement ratio, and their ranges are summarized in Table 3. Figure 3 shows a graphical representation of the laser ablation track and line scanning strategy. D, SR, and MRR were used as output responses to evaluate the micromachining performance with the addition of GnP reinforcement particles. The objective was to increase the ablation depth and material removal rate and minimize the roughness of the fabricated microchannel surface.

Statistical analysis-based ANOVA was used to evaluate the graphene reinforcement ratio, scanning speed, and frequency effects on surface roughness, material removal rate, and channel depth. The coefficient of determination (R^2^) was used to evaluate the accuracy of the models. The statistical analysis was performed using the software Minitab 17.

### 2.4. Integrated Intelligent ANFIS–MOPSO Method

Multi-objective optimization determines the combination of input process parameter settings that jointly optimize a single response or multiple responses. Joint optimization must satisfy the requirements for all the output responses, measured using the integrated hybrid method and desirability method, i.e., evaluating how well a combination of predicted graphene reinforcement ratio, scanning speed, and pulse frequency satisfy the objective for the responses. To improve the optimization method, this work developed an integrated intelligent method-based ANFIS with a MOPSO approach for multi-objective optimization of GnP ratio and micromachining parameters. Thus, this method was used to build prediction models for monitoring the quality of channels and optimizing the micromachining parameters and GnP reinforcement ratio.

#### 2.4.1. Adaptive Neuro-Fuzzy Inference System Modeling Procedure

**Figure 4:**
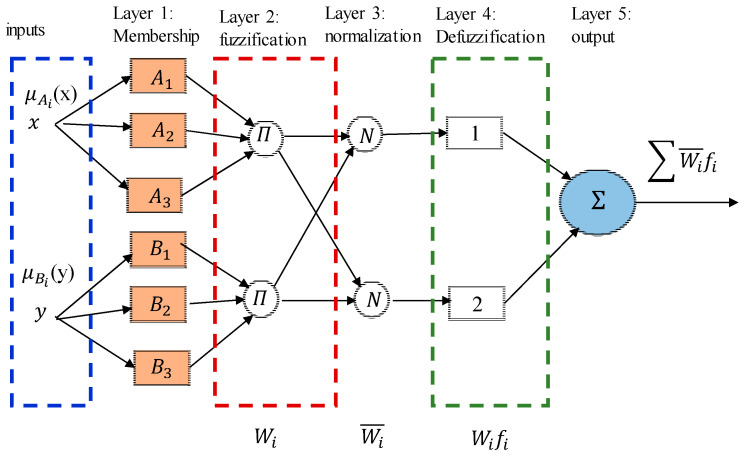
Architecture of ANFIS model.

ANFIS is a powerful tool for constructing prediction models for solving nonlinear and complicated problems. It combines fuzzy inference systems (FIS) and artificial neural networks (ANN). There are five layers in FIS, each with several node functions. For each input and output, the FIS structure has three membership functions (MFs), as illustrated in Figure 4. Each layer in the FIS is discussed in [58].

This work used ANFIS models to develop a nonlinear relationship between the inputs (GnP reinforcement ratio, scanning speed, and frequency) and the output responses (surface roughness, material removal rate, and channel accuracy). These models were developed to monitor the micromachining characteristics and then used as the fitness function for MOPSO to perform the optimization procedure. The experiments were divided into two groups to calculate the weights of each layer in FIS: the training group was used to train the model and the validation group, which includes the rest of the experiments was used to measure the accuracy of the ANFIS models. The ANFIS was developed using MATLAB 2020a.

#### 2.4.2. Multi-Objective Particle Swarm Optimization Approach (MOPSO)

MOPSO is a metaheuristic optimization algorithm that has been widely used to solve multi-objective optimization problems. MOPSO has been successfully applied to a wide range of MOOPs, including engineering design [59] and financial portfolio optimization [60]. In particular, MOPSO has been shown to perform well in comparison to other multi-objective optimization algorithms, such as the non-dominated sorting genetic algorithm (NSGA-II) [59] and the strength Pareto evolutionary algorithm (SPEA2).

**Figure 5:**
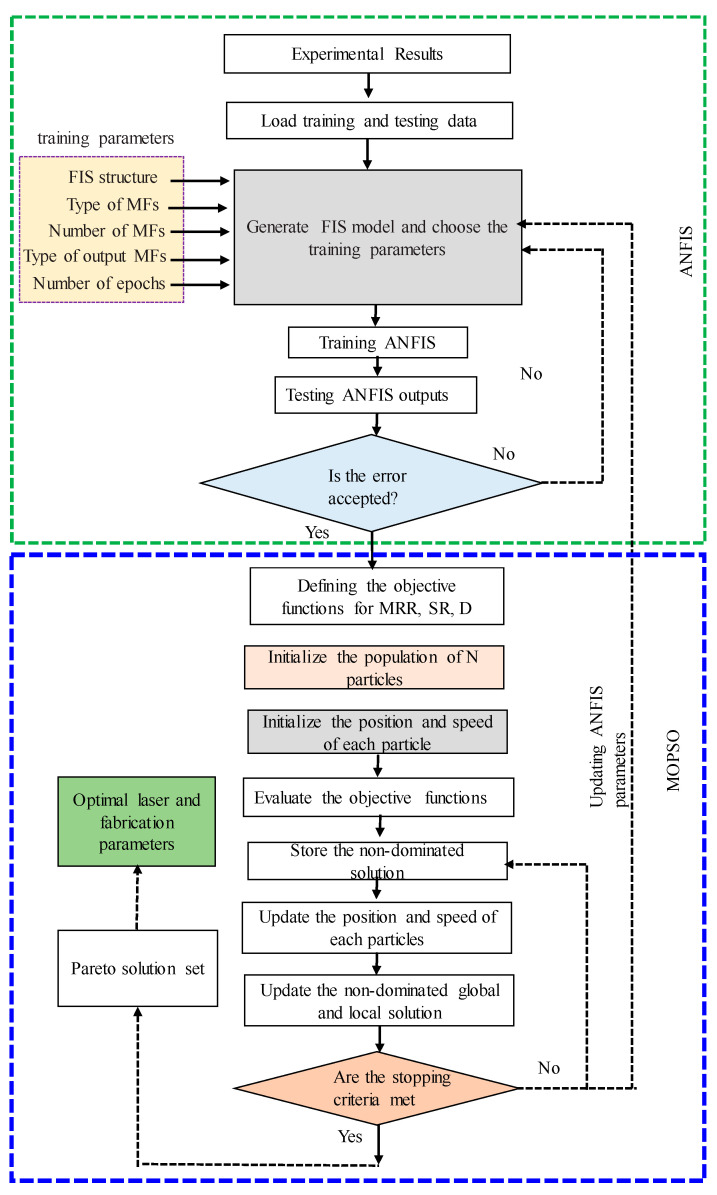
Structure of the integrated intelligent ANFIS–MPOSO method.

MOPSO’s accuracy depends on the appropriate formulation of fitness functions [61]. Thus, it is essential to correctly map the input values to the output values using the fitness function. Unfortunately, traditional methods often fail in the local solution when mapping nonlinear and complicated processes such as laser micromachining. Therefore, ANFIS techniques will be effective tools for developing models for complex nonlinear systems as a fitness function for the MOPSO approach. The structure overview of the proposed approach is shown in Figure 5. The details of the intelligent method for monitoring and optimizing the GnP ratio and laser parameters are as follows:

Step 1: Run the experiments and select the critical micromachining parameters. ANOVA testing is used to determine them.

Step 2: Divide the experiment data into the training and testing sets, then identify the initial parameters for the FIS structure.

Step 3: Train the ANFIS models for each output response based on the selected initial parameters for FIS. The FIS parameters are FIS structure, type of MFs, number of MFs, type of output MFs, number of epochs, and optimization methods. A minimum MAPE is obtained by updating the training parameter. Then, use the validating dataset to verify the developed model until all output responses are predicted accurately.

Step 4: Define the objective functions and constraints for all outputs according to the developed ANFIS models in the previous step.

Step 5: To execute MOPSO optimizations, it must identify MOPSO parameters. A set of MOPSO parameters is updated for the algorithm until it shows good convergence characteristics. Then, a 3D Pareto solution set is used to assess the optimal solution. In addition, MOPSO results are improved by updating ANFIS model parameters.

Step 6: To determine the optimal parameter set that meets the purpose of this study, evaluate the Pareto solution set obtained.

## 3. Results and Discussion

Table 4 presents all the combinations of the laser parameters and GnP reinforcement ratios based on the full factorial design and the corresponding output responses.

### 3.1. Statistical Analysis

Based on ANOVA with a 95% confidence interval, the effect of microlaser parameters and GnP reinforcement ratio was evaluated on the selected output responses. The *p*-value was used to determine whether the parameters and their interactions were statistically significant. An ANOVA model was built for each output response by initially employing all terms. Later, insignificant factors and their interactions in the models were removed using the backward elimination method (*p*-values not highlighted in Table 5 are more than 0.05). Subsequently, the ANOVA was performed again for the reduced models. Table 5 summarizes the ANOVA results for all outputs after eliminating the insignificant terms using backward elimination. Table 5 shows that all the terms with *p*-values less than 0.05 significantly affect D, SR, and MRR.

The coefficient of determinations for both models (R-squared adjusted and R-squared predicted) are presented in Table 6. It can be concluded that the models are adequate.

**Figure 6:**
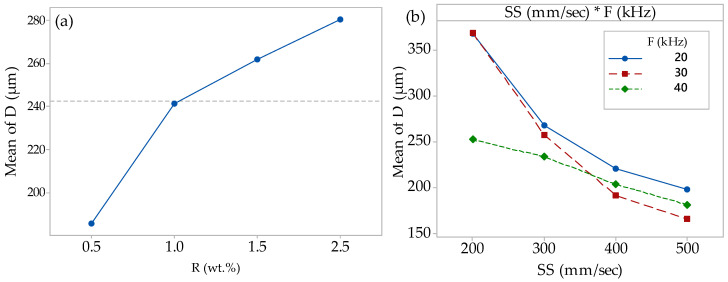
(**a**) Main effects for D, (**b**) interaction plot for D. (Note: × = * in all Figures).

The main effect plots for ablation depth are shown in Figure 6. It can be noted from the figure that the GnP reinforcement ratio affected the ablation depth. With the increasing GnP reinforcement ratio, the ablation depth increased. This is due to the enhanced thermal conductivity and optical absorbance of the developed material lowering the ablation threshold due to the addition of GnPs.

**Figure 7:**
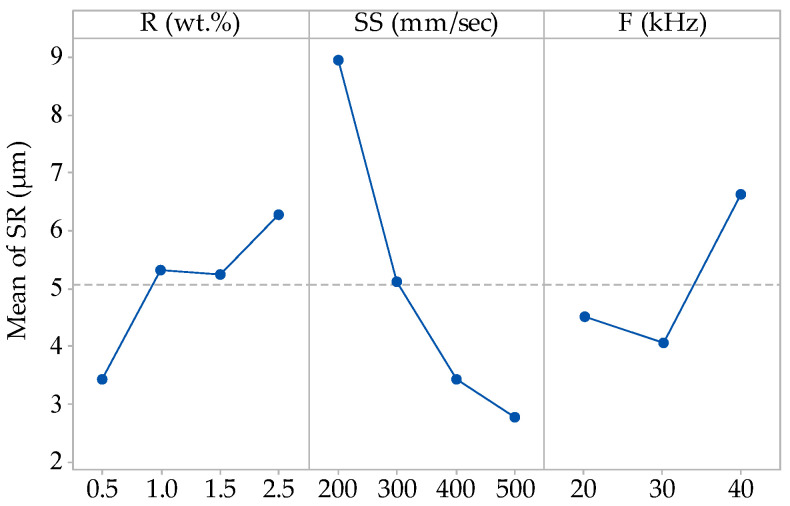
Main effects plot for SR.

**Figure 8:**
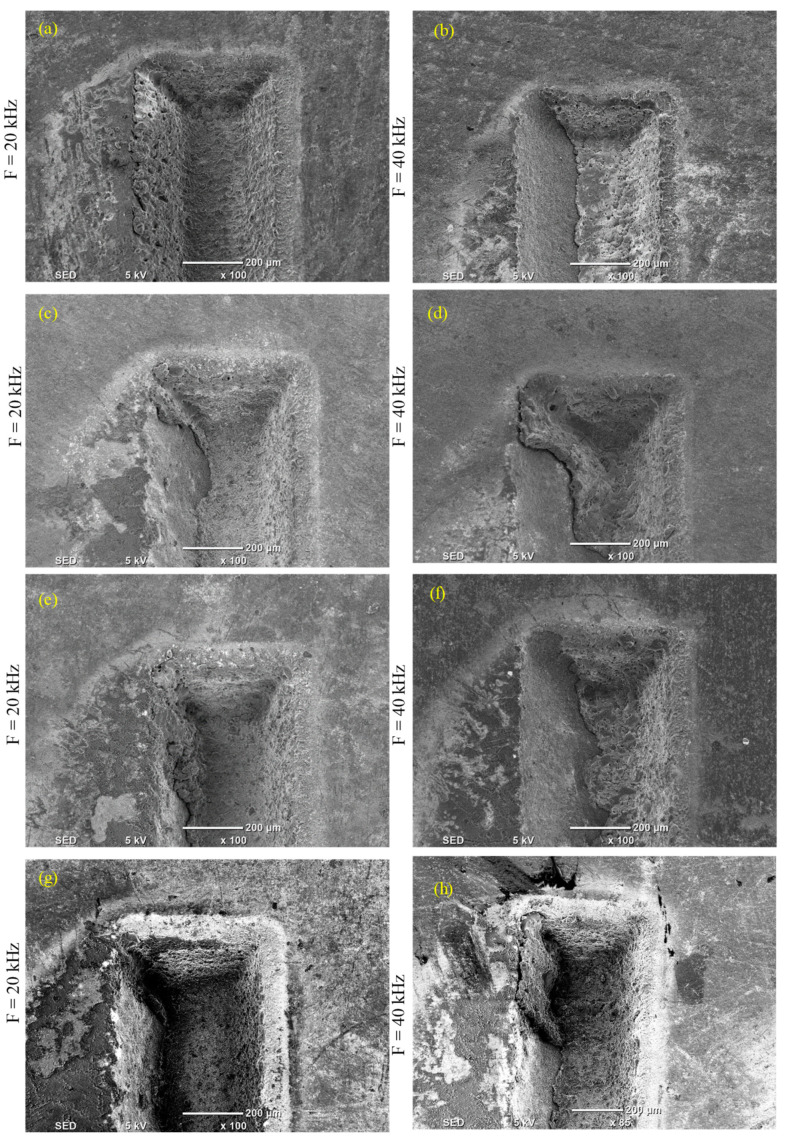
Surface morphologies with different GnP contents at a scanning speed of 300 mm/s. (**a**,**b**) 0.5 wt.% GnPs; (**c**,**d**) 1 wt.% GnPs; (**e**,**f**) 1.5 wt.% GnPs; (**g**,**h**) 2.5 wt.% GnPs.

The interaction effect plot in Figure 6 shows that increasing the scanning speed with frequency from 20 kHz to 30 kHz decreases the surface roughness. This is due to increasing the scanning speed with frequency, leading to a decrease the interaction time between the laser and materials, which leads to more evaporation and less debris re-deposition on the ablated surface [62]. From the main effects plots in Figure 7, it can be observed that the SR increased with the increasing GnP reinforcement ratio and decreased with the rising scanning speed. This is because more melted materials with increasing GnP content cannot be evaporated and removed from the bottom and sidewalls of the microchannel, forming the redeposited materials inside the channel. In addition, when increasing the frequency from 20 kHz to 40 kHz, the SR decreased and then increased with the increasing frequency to 40 kHz. Figure 8 shows typical images of the ablated microchannel and the influence of GnP contents and frequency on the SR and microchannel shape. It can be noted that the GnP contents and frequency have a significant impact on the quality of the produced microchannel. It can be seen from the SEM images that the microchannel shape changes with the increase in the frequency from 20 kHz to 40 kHz. Therefore, these changes affect the microchannel geometry. Thus, the calculated material removal rate depends on the geometry of the formed shape. In addition, from Figure 6, it can be seen that the microchannel depth decreased with increasing pulse frequency. However, the increase in MRR also results from the upper and lower width change.

**Figure 9:**
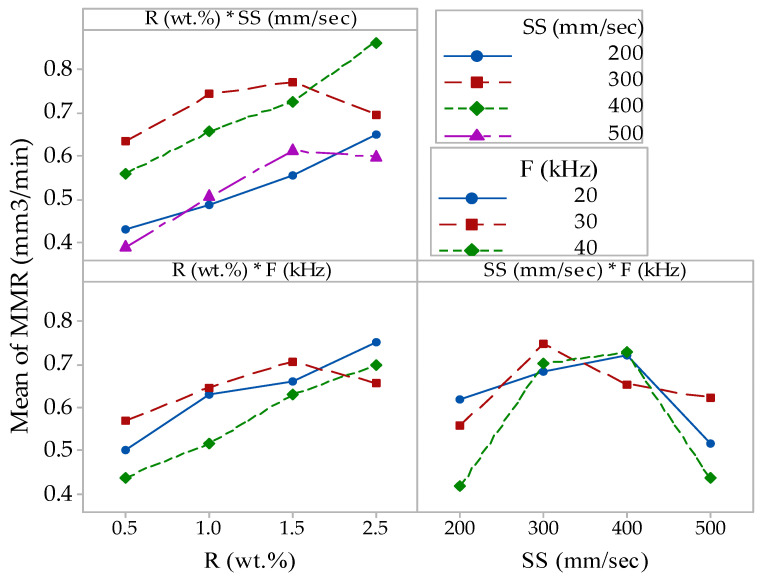
Main effect interaction plot for MRR.

From the interaction effect plot for MRR in Figure 9, it can be noted that the 1.2 wt.% GnP content and scanning speed of 400 mm/s obtained a higher MRR. In addition, the frequency of 30 kHz and the scanning speed of 300 mm/s also obtained a higher MRR.

### 3.2. Regression Models

Based on the design of the experiments, a mathematical model has been developed for D, SR, and MRR. The mathematical equations that best fit all of the selected responses are shown in the following equations.

(2) D(µm)=590.6+43.28R−0.968SS−7.83F+0.01580SS×F

SR (µm) = 9.00 − 0.02 R − 0.00810 SS − 0.0935 F − 0.00880 R × SS + 0.1451 R × F(3)

(4) $$MMR({mm}^{3}/min)=-0.499+0.2886R+0.006209SS-0.00328F-0.0628R\times R-0.000009SS\times SS$$

### 3.3. Predictive Model Development Based on ANFIS Technique

**Figure 10:**
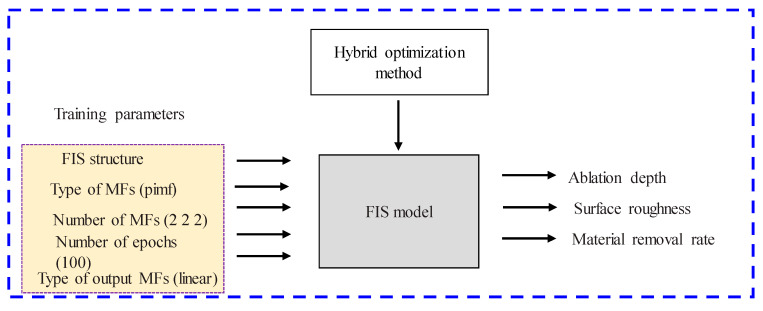
Initially selected parameters of adapted FIS algorithm for multiple outputs.

The ANFIS technique was used to develop models for monitoring and predictive micromachining outputs (D, SR, and MRR). The ANFIS models were evaluated for effectiveness based on the experimental results divided into training and testing data. Thus, training data were used to establish the models, while validating data were used to evaluate the developed models. In this study, the ANFIS technique was adapted for multiple outputs. Figure 10 shows the initially selected parameters for the ANFIS model.

After completing training, the FIS algorithm, the validating step, was performed to verify the effectiveness of the predictive ANFIS models. The MAPE was used to assess the performance of the developed ANFIS models, which can be computed using Equation (5).

(5) $$MAPE\frac{1}{n}\sum_{t=1}^{n}│\frac{{Expermental\;value}_{t}-{predicted\;value}_{t}}{{Experimental\;value}_{t}}│$$

where n is the number of training data. It should be noted that many fuzzy inference parameters were altered while the ANFIS algorithm was being trained to reduce MAPE. Table 7 shows the selected FIS parameters used to obtain the lowest MAPE.

**Figure 11:**
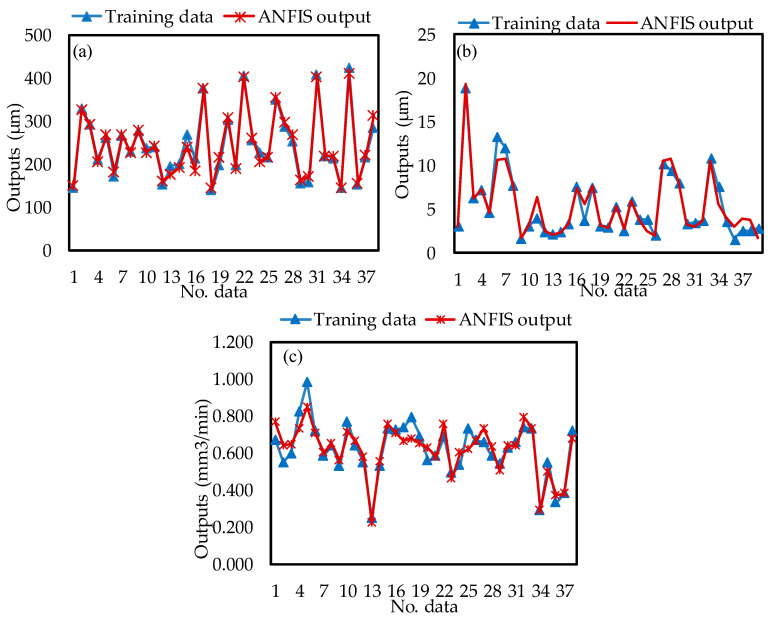
Comparison between the measured values and predicted ANFIS values (**a**) D; (**b**) SR; (**c**) MRR.

**Figure 12:**
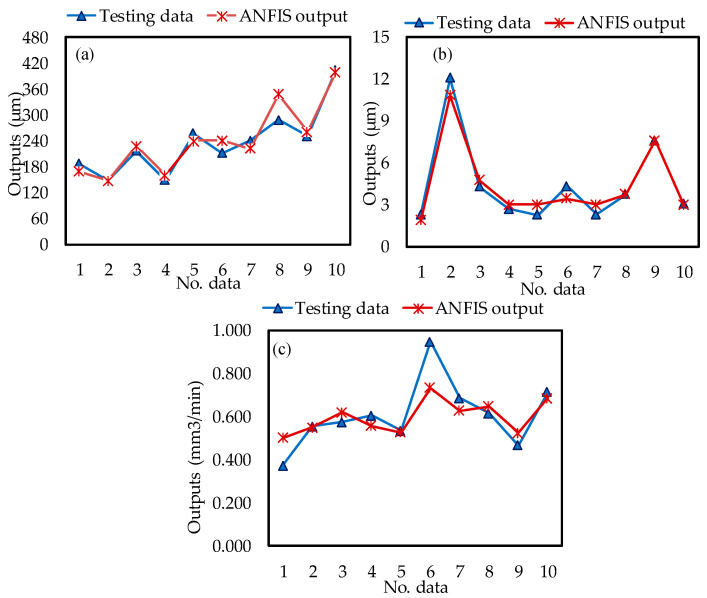
Comparison between the validating values and predicted ANFIS (**a**) D; (**b**) SR; (**c**) MRR.

Accordingly, the selected training parameters shown in Table 7 were used to conduct the FIS algorithm. The predicted ANFIS values for the investigated responses (D, SR, and MRR) were compared to the training and testing experimental values, which are shown in Figure 11 and Figure 12, respectively. The experimental and ANFIS predicted values are very close, indicating the accuracy of the constructed ANFIS models. Furthermore, it implies that robust ANFIS models can provide an accurate fitness function for the integrated MOPSO approach.

### 3.4. Comparison of the Artificial Intelligence Models with Regression Models

**Figure 13:**
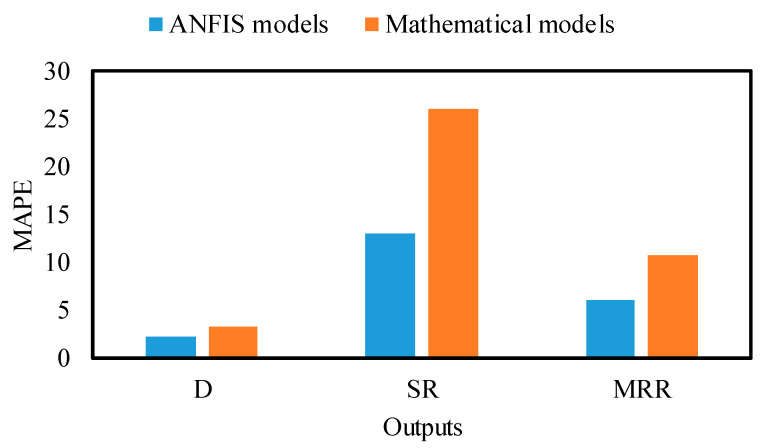
Comparison of the developed models.

For the micromachining response prediction, ANFIS and regression models were applied. The regression models for each response were developed using Minitab 17.0 software based on the full factorial design. Then, the values for each combination were generated using Minitab predictors. The MAPE of D, SR, and MRR for the two modeling techniques, ANFIS and regression models, are presented in Figure 13. A MAPE is an average of 48 experiments. It can be seen that the intelligence models performed better in estimating D, SR, and MRR compared with the mathematical models.

### 3.5. Multi-Objective Optimization Based on an Integrated Intelligent Method

Multiple responses were optimized to achieve a higher material removal rate, ablation depth, and minimum surface roughness. In this work, the material removal, ablation depth, and surface roughness responses are conflicting objectives. The objective is to maximize ablation depth and material removal rate simultaneously while minimizing surface roughness during micromachining GnPs/Al_2_O_3_ nanocomposites. It is, therefore, necessary to have a single set of GnP ratio and microlaser parameters (SS, F) for the optimal solution. In order to achieve this, multi-response optimization was conducted using integrated artificial intelligence with the MOPSO method based on the fitness functions developed using the ANFIS technique. Table 8 presents the optimal MOPSO parameters and micromachining constraints for optimization.

**Figure 14:**
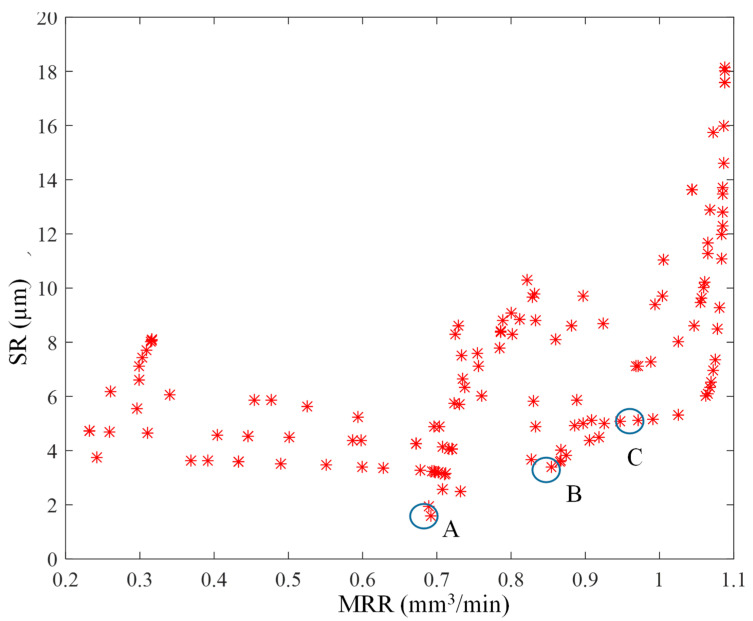
2D Pareto optimal solutions for SR and MRR.

Using the optimal parameters selected in the ANFIS and MOPSO algorithms, Figure 14 shows the Pareto optimal front. As shown in Figure 14, several potential solutions can be used simultaneously to optimize all outputs. Figure 14 shows three representative solutions (A–C) for micromachining parameters and the GnP ratio of alumina ceramic nanocomposites. A blue circle indicates the best solution, and a star circle indicates the non-dominated solution. Solutions at points A to C are optimal for D, SR, and MRR, as presented in Table 9.

### 3.6. Comparison with the Desirability Function Approach

The optimization of multiple responses was also conducted using desirability analysis to achieve higher D and MMR and minimum SR. The laser micromachining parameters and GnP reinforcement ratio were optimized using the desirability function based on developed regression models of the ablation depth, surface roughness, and material removal rate within the current range of experiment parameters. Furthermore, the performance of the integrated ANFIS–MOPSO approach is compared with the desirability approach. The optimal combination values of the laser micromachining parameters and GnP ratio that lead to maximum MRR and ablation rate and minimum SR based on the desirability method are shown in Table 10.

As shown in Table 10, to maximize D and MRR and minimize SR with 0.736 overall desirabilities, the scanning speed should be set at 200 mm/s, frequency at 20 kHz, and reinforcement ratio at 2.5 wt.%. The composite desirability of 0.736 indicates that the best outcome is achieved by combining the GnP reinforcement and laser parameters.

**Figure 15:**
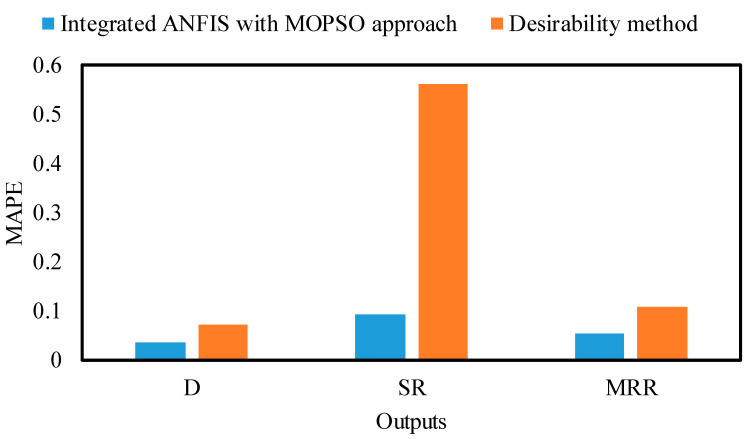
Evaluation of ANFIS–MOPSO and desirability method experiments using MAPE.

**Figure 16:**
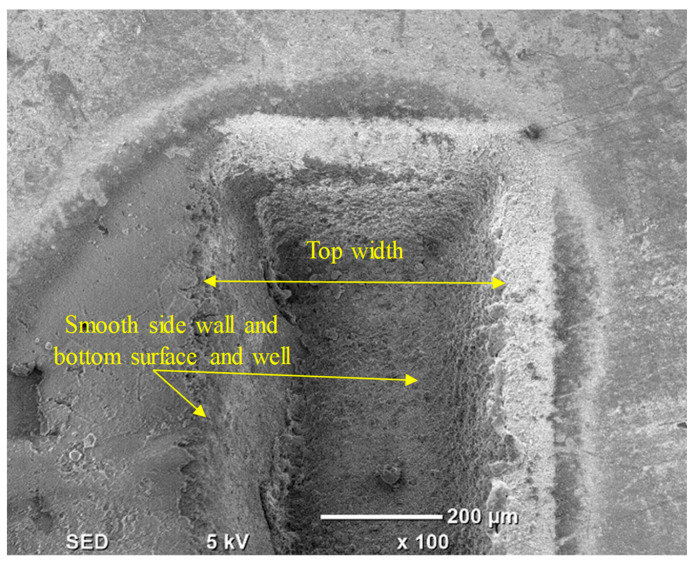
SEM picture of the fabricated microchannel using the optimized GnP reinforcement ratio and laser micromachining parameters.

The ANFIS–MOPSO and desirability multi-response optimization approaches were further validated by conducting additional experiments in optimal conditions. The validated experiments were repeated three times, and the average of the measured values was used. The comparison results are presented in Table 11. In addition, the MAPE was calculated, and is presented in Figure 15. It can be observed that the ANFIS–MOPSO method shows lower MAPE, which demonstrates the superior efficiency and effectiveness of this integrated intelligent method when compared to the desirability function method. In addition, Figure 16 shows an SEM picture of the fabricated microchannel using optimized GnP reinforcement ratio and laser micromachining parameters. It can be found that the optimized GnP reinforcement ratio and laser parameters produced higher microchannel quality with higher micromachining precision. In contrast, the unreinforced alumina ceramic could not be machined using the same optimized parameters using a low-power laser technique. This is because the graphene reinforcements reduce the ablation threshold of the Al_2_O_3_ and raise the removal efficiency due to higher optical absorbance and thermal conductivity as well as smaller grain size.

## 4. Conclusions

The reported studies in the literature on optimizing GnP ratio and microfabrication parameters to enhance the micromachining performance of monolithic Al_2_O_3_ are still rare and incomplete. To overcome this, GnPs/Al_2_O_3_ nanocomposites with varying GnP graphene contents of 0.5 wt.%, 1 wt.%, 1.5 wt.%, and 2.5 wt.% were successfully produced using ball mill powder processing and the HFIHs process. Afterward, micromachining experiments were conducted using low-power laser technology to investigate the influences of graphene reinforcement ratio, scanning speed, and frequency on the D, SR, and MRR of the Al_2_O_3_ nanocomposites. After that, an integrated artificial method based on an ANFIS–MOPSO approach was developed to optimize the laser microfabrication processing and GnP reinforcement ratio to improve the quality of the fabricated microchannel and multi-objective optimization method. As a result, the following main conclusions can be drawn:

- According to the ANOVA results, it was found that the GnP reinforcement ratio, scanning speed, and frequency have a significant effect on D, SR, and MRR. In addition, the GnP reinforcement ratio shows prominent effects on micromachining quality.
- Artificial intelligence models based on ANFIS were successfully developed to monitor and predict the D, SR, and MRR during laser micromachining. The ANFIS models show superior prediction performance for micromachining characteristics compared with regression models. The results showed that the ANFIS model could accurately estimate the D, SR, and MRR with lower MAPE of 2.17%, 6.03%, and 13%, respectively, compared with experimental results.
- It was determined that a scanning speed of 342 mm/s, a frequency of 20 kHz, and a reinforcement ratio of 2.16 wt.% were the optimal combinations for laser micromachining parameters and the GnP reinforcement ratio. This resulted in a depth of 285.6 mm, MRR of 0.8531 mm^3^/min, and a surface roughness of 3.391 µm. These combinations can fabricate microchannels on alumina ceramics for a microfluidic device with higher quality and accuracy compared with unreinforced Al_2_O_3_. The existing nanocomposites are also green as they reduce energy consumption and create a clean environment, whereas the unreinforced alumina could not be machined by using the same optimized parameters using a low-power laser technique.

In a nutshell, this study demonstrates a huge potential for optimizing the GnP reinforcement ratio to improve the micromachining efficiency of alumina ceramics. Therefore, this can be extended further to silicon nitride, silicon carbide, and zirconia to enhance the material removal efficiency. In addition, the developed integrated ANFIS with MOPSO approach can be implemented for complex micromachining processes, including electric discharge machining, wire electric discharge machining, and µ-RUM.

## Figures and Tables

**Table 1:** Composition of Al_2_O_3_ powder.

Elements	Na_2_O	B_2_O_3_	CaO	Fe_2_O_3_	MgO	Al_2_O_3_
Percentage (wt.%)	≤0.03	≤0.002	≤0.01	≤0.01	≤0.02	balanced

**Table 2:** The Vickers hardness of the produced nanocomposite samples.

GnP Ratio (wt.%)	Hardness (HV)
0.5	1564 ± 7.4
1	1477 ± 9.4
1.5	1432 ± 6.7
2.5	1398 ± 8.6

**Table 3:** Micromachining parameters, GnP reinforcement ratio, and their selected levels.

Laser Parameters and GnPs Ratio	Values			
Scanning speed, SS (mm/s)	200	300	400	500
Pulse frequency, F (kHz)	20	30	40	-
Reinforcement ratio, R (wt.%)	0.5	1	1.5	2.5
Strategy	Line			

**Table 4:** Experimental design and corresponding results.

Exp. No.	R (wt.%)	SS (mm/s)	F (kHz)	D (µm)	SR (µm)	MMR (mm^3^/min)
1	0.5	500	20	145.33	2.59	0.373
2	0.5	200	40	186.71	3.06	0.288
3	0.5	400	40	147.00	2.53	0.554
4	1.5	500	20	217.61	2.67	0.574
5	2.5	200	40	329.25	18.95	0.549
6	1.0	500	30	148.31	1.59	0.604
7	0.5	200	20	292.24	6.33	0.534
8	2.5	400	20	257.22	4.31	0.946
9	0.5	300	30	211.33	2.30	0.685
10	2.5	500	30	210.25	2.85	0.613
11	1.0	200	40	240.40	12.06	0.331
12	0.5	200	30	288.17	7.26	0.465
13	1.0	300	40	250.56	7.59	0.713
14	1.0	500	40	171.07	3.03	0.380
15	1.0	300	20	267.27	4.67	0.720
16	2.5	300	40	262.12	13.29	0.671
17	1.5	200	30	404.14	7.74	0.547
18	2.5	500	20	229.02	1.71	0.596
19	1.5	300	30	276.88	3.13	0.820
20	2.5	400	40	237.51	3.99	0.983
21	1.5	400	20	241.011	2.46	0.717
22	0.5	400	30	152.27	2.15	0.586
23	1.0	400	30	196.14	2.44	0.641
24	1.0	500	20	200.33	3.41	0.526
25	1.5	300	40	268.00	7.59	0.764
26	1.0	400	40	212.80	3.71	0.635
27	1.0	200	30	377.13	7.50	0.547
28	0.5	500	40	140.22	3.13	0.248
29	1.5	500	40	199.37	2.98	0.529
30	2.5	300	20	302.24	5.32	0.730
31	1.5	400	30	197.71	2.62	0.726
32	2.5	200	20	406.58	5.95	0.735
33	1.0	300	30	256.32	3.93	0.793
34	1.0	400	20	228.33	3.83	0.689
35	0.5	300	20	217.33	2.01	0.558
36	1.0	200	20	352.16	10.27	0.581
37	2.5	300	30	288.17	4.27	0.685
38	1.5	200	40	254.51	9.45	0.491
39	0.5	400	20	155.26	2.30	0.533
40	1.5	500	30	160.44	2.25	0.731
41	2.5	200	30	408.78	8.06	0.667
42	2.5	400	30	220.33	3.30	0.659
43	2.5	500	40	214.21	3.53	0.583
44	0.5	500	30	145.14	3.73	0.542
45	1.5	200	20	424.23	10.90	0.624
46	0.5	300	40	154.15	3.73	0.658
47	1.5	400	40	217.11	7.59	0.736
48	1.5	300	20	286.27	3.57	0.729

**Table 5:** ANOVA results for output responses.

Output	Factors and Their Interaction	*p*-Value
	Model	**0.00**
	R	**0.00**
	SS	**0.00**
	F	**0.00**
Depth	2-Way Interactions	**0.00**
	R × SS	0.095
	SS × F	**0.00**
	R (wt.%)	**0.018**
	SS (mm/sec)	**0.00**
Surface roughness	F (kHz)	**0.004**
	2-Way Interactions	0.167
	R × SS	0.379
	R × F	0.084
	R (wt.%)	**0.00**
	SS (mm/sec)	**0.00**
	F (kHz)	**0.001**
MRR	2-Way Interactions	**0.00**
	R (wt.%) × SS (mm/s)	**0.006**
	R (wt.%) × F (kHz)	**0.012**
	SS (mm/sec) × F (kHz)	**0.00**

**Table 6:** Model accuracy parameters for output responses.

Output	R-Squared (Adjusted)	R-Squared (Pred)
D	95.82	91.46
SR	66.2%	31.1%
MRR	89.49.2%	71.38%

**Table 7:** Selected FIS parameters.

Output	D	SR	MRR
Training optimization method	Hybrid method		
MF type	psigmf	trimf	gbellmf
Number of MFs	2 3 3	2 3 2	2 3 2
Number of epochs	200	600	800
Output function type	constant	constant	constant

**Table 8:** MOPSO Parameters and microfabrication constraints.

Parameters	Values
Size of Population	50
Number of Iterations	160
Inertia Weight (w)	0.4
Personal Learning Coefficient (C1)	0.8
Global Learning Coefficient (C2)	1.5
Microfabrication Constraints	200 ≤ SS ≤ 500 mm/s 20 ≤ F ≤ 40 kHz 0.5 ≤ R ≤ 2.5 wt.%

**Table 9:** Optimal laser micromachining parameter and GnP reinforcement ratio.

Solution	SS (mm/s)	F	R (wt.%)	D (µm)	SR (µm)	MRR (mm^3^/min)
A	388	20	0.66	182.0	1.7	0.6
B	342	20	2.16	285.6	3.3	0.8
C	339	21	2.5	273.8	5.1	0.97

**Table 10:** Optimal laser micromachining parameters and GnP ratio obtained through desirability approach.

SS (mm/s)	F (kHz)	R (wt.%)	D (µm)	SR (µm)	MRR (mm^3^)	Desirability
200	20	2.5	419.4	9.0	0.77	0.73
300	30	1.5	281.2	3.4	0.8	0.70
400	20	2.5	253.3	1.8	0.9	0.71

**Table 11:** Validation experiments of ANFIS–MOPSO and desirability methods.

Parameters	SS (mm/s)	F (kHz)	R(wt.%)	D (µm)	SR (µm)	MRR (mm/min)
ANFIS–MOPSO	339	20	2.5	273.8	5.115	0.97
Experimental	339	20	2.5	283.69	4.681	0.92
Desirability Approach	200	20	2.5	419.46	9.01	0.77
Experimental	75	90	1.2	391.09	5.95	0.69

## Data Availability

Data are included within the article.
